# Unveiling the hidden burden: estimating the proportion of undiagnosed depression, hypertension and diabetes – a modelling study using survey data from adults in England, 2011–2019

**DOI:** 10.1136/bmjph-2024-001919

**Published:** 2025-10-30

**Authors:** Benjamin Barr, Anna Head, Brendan Collins, Chris Kypridemos

**Affiliations:** 1 Public Health, Policy and Systems, University of Liverpool, Liverpool, UK

**Keywords:** Public Health, Health Services Accessibility, Mental Health, Prevalence

## Abstract

**Background:**

A large proportion of chronic conditions are undiagnosed, preventing early treatment, and leading to poorer outcomes. Understanding how levels of underdiagnosis vary between diseases and population groups over time is crucial for effectively allocating resources and targeting interventions to increase diagnosis rates.

**Methods:**

We used two annual national surveys: the Health Survey for England (cross-sectional) and the UK Household Longitudinal Survey, to identify people with diabetes, hypertension and depression. Diagnosed cases were defined as a self-report of being told by a nurse or doctor as having a condition; undiagnosed cases were defined as those where screening tools used in the survey identified clinical signs of the condition but the individual did not self-report a diagnosis. We used logistic regression to estimate the proportion of people with these three conditions who are undiagnosed for 540 population segments defined by age group, sex, deprivation quintile and region between 2011 and 2019. These predicted probabilities were applied to population estimates using microsimulation to model the proportion undiagnosed for each disease in each Clinical Commissioning Group (local health planning areas) in England.

**Results:**

The proportion of people with diabetes and depression who were undiagnosed reduced between 2011 and 2019, with no change in the proportion of hypertensives undiagnosed. For hypertension, people in more deprived areas were less likely to be undiagnosed than those in less deprived areas. The opposite was true for depression. Younger men with hypertension or diabetes were less likely to be diagnosed than older men. Both those aged under 30 and those over 70 with depression were less likely to be diagnosed compared with those aged 30–70.

**Conclusion:**

Strategies aiming to improve undiagnosed hypertension case finding need to understand the reasons for little progress over the past decade. For depression, strategies to increase early diagnosis should prioritise deprived areas. Case finding for all three diseases would benefit from targeting younger age groups.

**WHAT IS ALREADY KNOWN ON THIS TOPIC**

- There have been some previous studies estimating the proportions of people with diabetes, hypertension and depression who are undiagnosed.
- We do not know how this varies over time, between sociodemographic groups (age, sex, deprivation) or between places.

**WHAT THIS STUDY ADDS**

- This study shows how access to diagnosis for depression and diabetes has improved over the past decade, while undiagnosed hypertension case finding appears to have stalled.
- Groups with a lower probability of diagnosis include younger age groups and deprived populations in the case of depression.
- More affluent groups have a lower chance of being diagnosed with hypertension when they have clinical signs of the disease.

**HOW THIS STUDY MIGHT AFFECT RESEARCH, PRACTICE OR POLICY**

- Further investigation should seek to understand the lack of improvement in hypertension case finding over the past decade to inform the current National Health Service strategy.
- Actions to increase case finding for all diseases should target those populations with higher levels of underdiagnosis.
- For depression, this would help reduce mental health inequalities, while for hypertension and diabetes, we find no evidence for inequalities in underdiagnosis.

## Introduction

 The timely diagnosis of chronic conditions is crucial for initiating early treatment and minimising mortality and complications.^1^ However, research suggests that a significant portion of chronic conditions remains undiagnosed even in countries with accessible healthcare services. For instance, in England, estimates reveal that approximately 30% of cases of hypertension and diabetes mellitus^2 3^ (henceforth referred to as diabetes) and 36% of depression^4^ go undetected.

Underdiagnosis, defined as the proportion of undetected cases of a disease, has been linked to factors such as age, gender and socioeconomic status.^5^ Underdiagnosis can result from patient and health system factors. For example, patients may not be diagnosed or may be diagnosed late if they lack awareness of signs, symptoms and risk factors, experience stigma or mistrust the healthcare system. Alternatively, health systems may not be adequately accessible, where services are not available, culturally appropriate, easy to get to or affordable.^6^ These disparities in healthcare access and utilisation pose challenges for public health and exacerbate health inequalities but also impose considerable burdens on healthcare systems.^1^

Many countries employ geographical funding formulas based on diagnosed conditions to allocate public healthcare funds to different regions.^7^ However, if certain population groups are less likely to receive a diagnosis, formulae just based on diagnosed conditions may inadequately address the healthcare needs of those groups, potentially leading to discrimination. Thus, understanding variations in underdiagnosis rates among diseases and population groups is essential for equitable resource allocation and targeted interventions to improve diagnosis rates.

Quantifying underdiagnosis presents challenges as it is unobservable in healthcare data. However, for conditions like diabetes, hypertension and depression, diagnostic tests and screening tools exist and are often incorporated into health surveys alongside self-reports of diagnosed conditions, allowing calculation of estimates of total disease prevalence and the fraction that remains undiagnosed. Previous approaches have relied on small-area modelling using a combination of national survey data and census measures to estimate small area disease prevalence that is then compared with diagnosed prevalence from primary care records.^8^,^11^ These may quickly become outdated, as censuses are only carried out decennially, and bias may be introduced by comparing patterns of diagnosis and disease from different data sources (survey data and primary care records). An alternative method that has not previously been implemented is to estimate the probability of diagnosis for population segments, directly using survey responses related to self-reported clinical diagnoses and probable disease indicators as measured in survey screening tools. These can be used to estimate underdiagnosis for small areas.

To address this gap in understanding, our study aimed to use this approach to estimate the fraction of undiagnosed cases of hypertension, diabetes and depression in England between 2011 and 2019 and how this varied across population demographics and geographical areas.

## Methods

### Data sources and measures

We estimated the fraction of people with diabetes, hypertension and depression who were undiagnosed for each age and sex group, within five quintile groups of deprivation (deprivation quintile) based on the index of multiple deprivation (IMD)^12^ score of the neighbourhood in which patients lived, within each of the nine geographical regions of England (North West, North East, Yorkshire and the Humber, West Midlands, East Midlands, East of England, London, South East, South West) for each year between 2011 and 2019 using nationally representative annual survey data. Five age groups were defined (18–29, 30–49, 50–59, 60–69, 70–79, 80+). We focus on these three conditions because they are the only chronic conditions for which there are screening tools for clinical signs implemented in regular nationally representative surveys in England enabling a comparison between self-reported diagnosis and a survey-based measure of clinical symptoms.

For diabetes and hypertension estimates, we use annual data from the Health Survey for England (HSE)^13^ between 2011 and 2019. HSE is an annual cross-sectional nationally representative survey collecting detailed information on mental and physical health conditions, including biological measures, alongside demographic and socioeconomic characteristics of people aged 16 years and over at private residential addresses.^14^ Data collection involves a face-to-face interview followed by a nurse visit from a random sample of households where blood pressure (three readings at 1 min intervals) and a non-fasting blood sample are taken. Each annual survey consists of a new cross-sectional sample, with household response rates varying between 59% and 66% between 2011 and 2019.^15^ Further details of the survey design are reported elsewhere.^14^

We defined people as having clinical signs of hypertension if they have systolic blood pressure >140 mm Hg and clinical signs of diabetes if their blood test indicated glycated haemoglobin (HbA1c) ≥48 mmol/mol as measured during the nurse visit.^16^ People were defined as having been diagnosed with diabetes or hypertension if they responded ‘yes’ to the survey questions about ever having been told by a doctor or nurse that they have diabetes or hypertension, respectively. HSE does not distinguish between diabetes type 1 and type 2 in either self-report or clinical measurements. The total sample available for analysis was 35 669 between 2011 and 2019; we used nurse and interview survey weights to account for differences in response rates.

For depression estimates, we used annual data from the Understanding Society UK household longitudinal study (UKHLS).^17^ This is a nationally representative, annual longitudinal panel survey based on a stratified clustered random sample of 40 000 households from the four UK countries. Data are collected by trained interviewers using face-to-face surveys. Everyone in the sampled households aged 16 and over completed a questionnaire covering a range of dimensions, including the Short-Form Health Survey (SF-12). Response rates of 62% were achieved in the initial sample, and reinterview response rates have generally been between 70% and 90% (2011—78%, 2019—86%). Further details of the survey design are reported elsewhere.^18^ We used longitudinal weights to correct for unequal selection probability, lack of response in the first wave and attrition in the following waves. The total sample available for analysis was 179 210 person-years (52 142 people) over the nine study years.

We defined people as having clinical signs of depression if during the face-to-face interview they scored ≤42 on the Mental Component Summary (MCS) of the SF-12, which has been found to reflect clinical levels of depressive symptoms.^19 20^ People were defined as having been diagnosed with depression if they reported having ever been diagnosed with depression in the initial wave they entered the survey or if they reported being diagnosed with depression in subsequent waves (see online supplemental Appendix 1 and 2 for further details).

A summary of definitions of diagnosed cases and clinical signs for each condition is provided in table 1. Within our study, we define ‘diagnosed’ cases as when an individual self-reported having been told they had a condition; and ‘undiagnosed’ is when an individual had clinical signs of a condition without a self-report of being told they had the condition.

**Table 1:** Summary of measures for diagnosed cases and clinical signs of depression, hypertension and diabetes

Condition	Data source	Diagnosed case definition based on self-report of condition	Clinical signs during study visit
Depression	Understanding Society UK household longitudinal study (UKHLS)	Self-report of ever having been diagnosed with depression	≤42 on the Mental Component Summary (MCS) of the Short-Form Health Survey (SF-12) at study visit
Diabetes	Health Survey for England (HSE)	Self-report of ever having been told they have diabetes by a nurse or doctor	HbA1c ≥48 mmol/mol from blood test taken by nurse during study visit
Hypertension	Health Survey for England (HSE)	Self-report of ever having been told they have hypertension by a nurse or doctor	SBP >140 mm Hg as measured by nurse at study visit

HbA1c, glycated haemoglobin; HSE, Health Survey for England; SBP, systolic blood pressure.

### Analysis

For each disease, we fitted a logistic regression model estimating the probability of people not having a diagnosis conditional on having the disease, where the outcome was an indicator of not reporting being diagnosed, with the data set filtered only to include those with disease (either clinical signs of disease or a diagnosis of disease). As predictors in these models, we included all two-way interactions between age group, deprivation quintile, sex, region and time period as a linear trend term (see online supplemental appendix 4 for further details).

We limit our models to only include these variables as these are the only variables available in the surveys that are also available for small area population estimates to enable microsimulation. Using this limited set of variables also provides sufficient survey sample size within each population segment. Interactions are included to capture heterogeneity in unmet needs across these groups (age, sex, region, deprivation). For example, it is plausible that because of differences in health systems and population composition between regions, the relationship between deprivation and underdiagnosis varies by region.

We used stepwise model selection by Akaike information criterion (AIC) to find the combination of interactions that provides the best fitting model for the data.^21^ This final model was then used to predict the probability of having the disease and the probability of not being diagnosed conditional on having the disease for all intersections between age group, deprivation quintile, sex, region and time period. To aid the interpretation of the model outputs,^22^ we plotted the mean of the adjusted predictions for the probability of being undiagnosed across age groups, sex and deprivation quintiles for 2011 and 2019, using the *marginaleffects* package in R.^23^

We applied these estimates to population segments in 2011 and 2019 using a simple microsimulation approach to produce estimates of the fraction of people with disease who were undiagnosed for each of the 191 health planning areas (Clinical Commissioning Groups (CCGs)). We first used the Office for National Statistics mid-year population statistics^24^ to construct a microdata set for all∼38 million people in England 2011 and 2019, for each age group (18–29, 30–49, 50–59, 60–69, 70–79, 80+) and sex (male/female), within small neighbourhoods (Lower Super Output Areas), with their associated level of deprivation (IMD quintile), nested within CCGs, that lie within nine English Regions. We then estimated a set of flags indicating whether individuals had each of the three conditions. This initially involved estimating a similar logistic regression model as outlined above; however, in this case, the main outcome was the probability of having either a diagnosis or clinical signs (1) or having no diagnosis or clinical signs (0). Based on the predicted probability from this model, we create a flag (0/1) in our population microdata set drawn from a binomial distribution indicating whether each individual in the population has each of the three conditions. Then, based on the predicted probability from our main model indicating the probability of being undiagnosed (conditional on having the condition), we create an additional flag (0/1) drawn from a binomial distribution indicating whether each person with disease is undiagnosed (1) or diagnosed (0). Flags for each condition were drawn from independent binomial distributions. These were then aggregated for each CCG, giving an estimate of the proportion of cases undiagnosed for each CCG (see online supplemental Appendix 7 for further details).

## Sensitivity analysis

While the SF-12 MCS has been used to screen populations for mental health conditions, it does not provide a precise diagnostic definition for depression. We, therefore, repeated the analysis using a lower threshold on the SF-12 MCS to define clinical signs of depression (see online supplemental Appendix 6). As an alternative measure of levels of underdiagnosis that combined survey-based data with primary care clinical records, we estimated the fraction undiagnosed in each CCG, comparing the overall survey-based estimated prevalence of disease with the diagnosed prevalence of disease for each CCG as reported through the Quality and Outcomes Framework (QOF), a national reporting scheme for general practitioner (GP) practices^25^ (see online supplemental Appendix 6).

## Results

A summary of participant characteristics from HSE and Understanding Society is presented in online supplemental Appendix 3. The full output for each logistic regression model is given in online supplemental Appendix 4. Hosmer-Lemeshow Goodness-of-Fit tests indicate the models were a good fit to the data. For diabetes, we estimated from these models that for 2019, 22% (95% CI 19.5% to 25.1%) of cases in England were undiagnosed, decreasing from 26% (95% CI 23.4% to 28.5%) in 2011. In the regression model, none of the parameters were statistically significant at the 5% level, and only interactions between year and deprivation, and age group and sex improved the model fit based on the AIC and therefore remained in the final model (see online supplemental Appendix 4).

**Figure 1:**
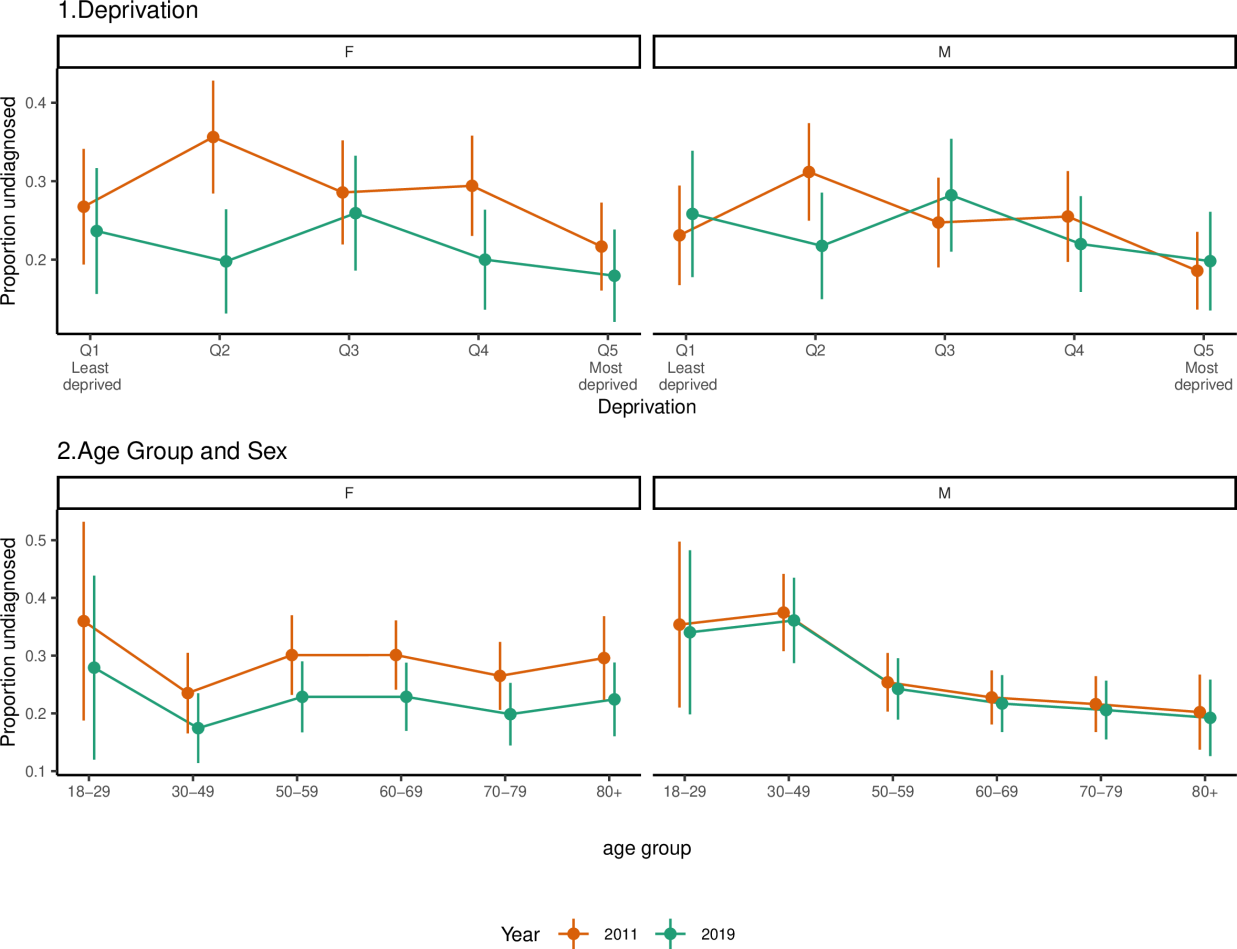
Mean predictions of the probability of people with diabetes being undiagnosed by deprivation, age and sex in 2011 and 2019, from the logistic regression models, showing that levels of underdiagnosis did not vary markedly with deprivation and sex, with men under 50 tending to have slightly higher levels of underdiagnosis. F, female; M, male.

Figure 1 shows the mean of the adjusted predictions from this model for the proportion of diabetes cases undiagnosed by deprivation, age and gender for 2011 and 2019. This indicates that levels of underdiagnosis did not vary markedly with deprivation, with slightly better levels of diagnosis in the more deprived areas. Underdiagnosis did not vary markedly by age group for women; however, men under 50 tended to have slightly higher levels of underdiagnosis.

**Figure 2:**
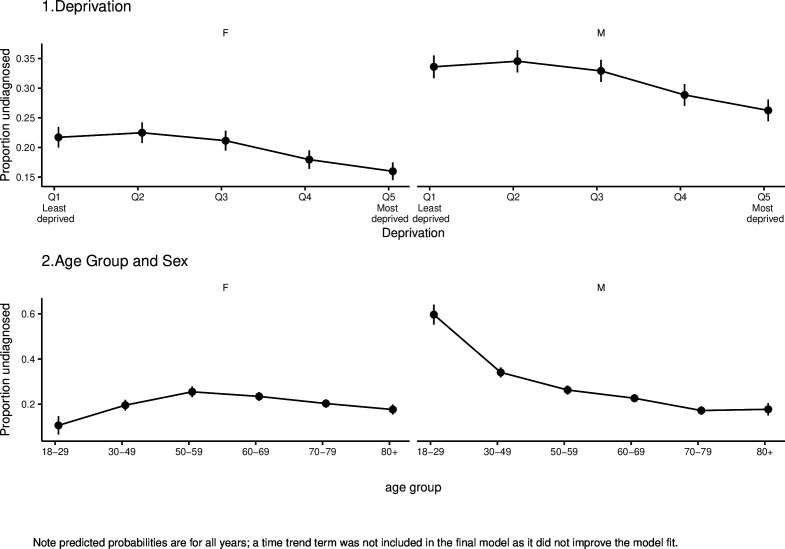
Mean predictions of the probability of people with hypertension being undiagnosed by deprivation, age and sex 2011−2019, from the logistic regression model, showing higher underdiagnosis for men, declining underdiagnosis with increasing level of deprivation and varying age patterns by sex. F, female; M, male.

For hypertension, we estimate that 23% (95% CI 21.8% to 23.2%) of people with hypertension in England were undiagnosed between 2011 and 2019, and this had not changed over time (figure 2). The model fitting process found that including a time trend for year did not improve the model fit, so this was removed from the final model. The only interactions that were included in the final model were age group-sex interactions. These findings are shown in figure 2 which indicates that levels of hypertension underdiagnosis were higher for men compared with women and declined with increasing levels of deprivation. The relationship with age varied by gender, with underdiagnosis for men highest at younger ages and declining markedly at older ages. For women, the highest levels of underdiagnosis are between ages 50 and 59 years old, with lower levels in young and older age groups.

**Figure 3:**
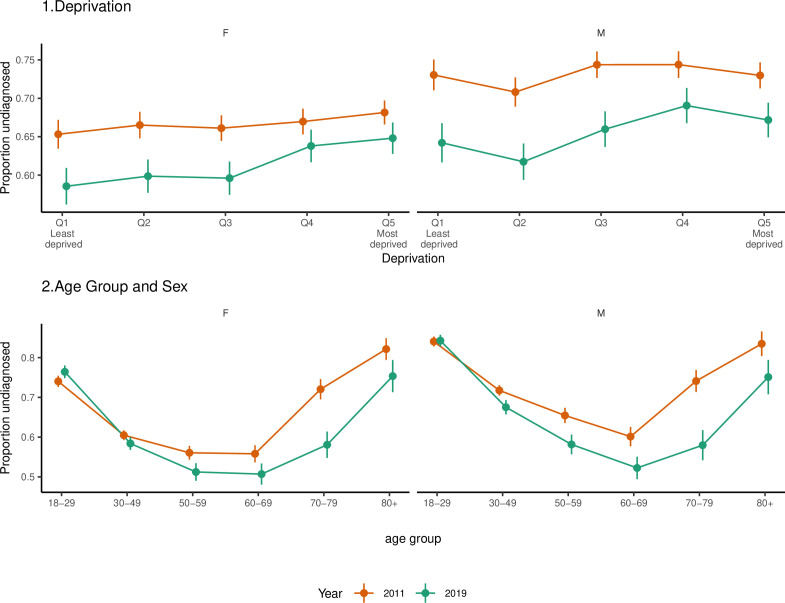
Mean predictions of the probability of people with depression being undiagnosed by deprivation, age and sex in 2011 and 2019, from the logistic regression model, showing levels of underdiagnosis of depression reduced over time, were higher for men, increased with increasing levels of deprivation and were highest for younger and older age groups. F, female; M, male.

For depression, we estimate that in 2019, 64% (95% CI 63% to 65%) of people with clinical signs of depression in England were undiagnosed, decreasing from 68% (95% CI 67% to 68%) in 2011. The final regression model included all two-way interactions. Figure 3 shows the adjusted predictions from this model for the proportion of people with depression who were undiagnosed by deprivation, age and gender for 2011 and 2019. Levels of underdiagnosis of depression were higher for men compared with women and increased with increasing levels of deprivation, particularly for women. The probability of diagnosis has increased over time but to a greater extent for the least deprived and older age groups. We see a clear U-shaped relationship with age, with the highest levels of underdiagnosis in the youngest and oldest age groups. The relationship between underdiagnosis of depression and deprivation varied considerably between age groups and regions (see online supplemental Appendix 4).

**Figure 4:**
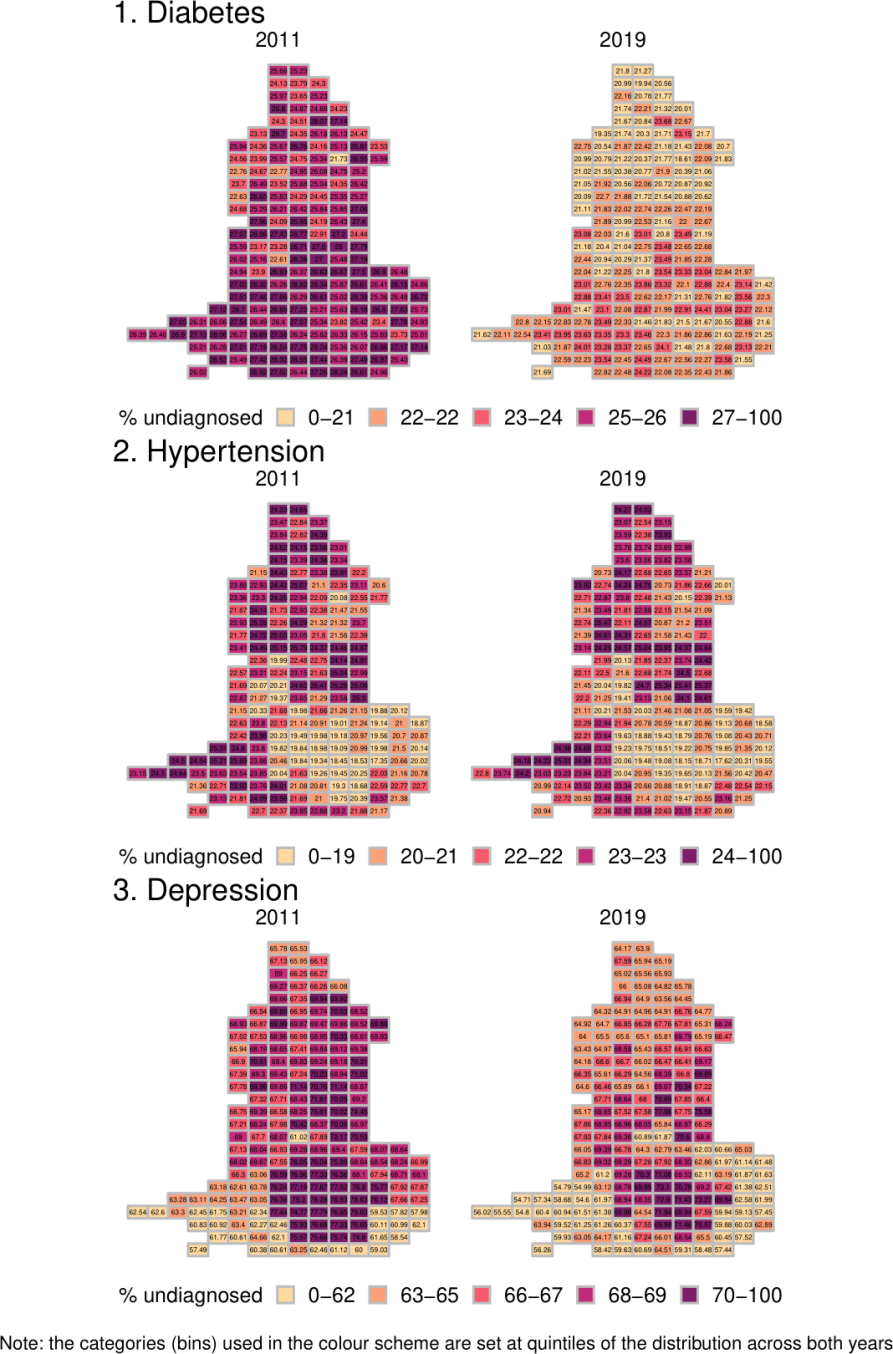
Estimates percentage of conditions undiagnosed by CCG (darker colour indicates higher % undiagnosed). CCG, Clinical Commissioning Group.

Figure 4 shows the estimates from the microsimulation. Most notable are the high estimated levels of underdiagnosis for depression in London and the low levels of underdiagnosis in the South West. For hypertension, we see lower levels of underdiagnosis in London, higher in the South West and the North. For diabetes, we see lower levels of underdiagnosis in the North and higher levels in the South. For diabetes and hypertension, we found little evidence to suggest that the geographical pattern of underdiagnosis has changed markedly over time (Morans I test for spatial correlation in the change in the % undiagnosed between 2011 and 2019, p=0.23 and p=0.11, respectively). For depression, the decreases in the % undiagnosed were greater in the southwest and outer London areas (Moran I test, p<0.001; see also online supplemental Appendix 5).

In sensitivity analysis, when we used a lower threshold on the MCS to define cases of probable depression, we saw a more marked increase in underdiagnosis with increasing deprivation (online supplemental Appendix 6). We compared the estimates above, which only used survey-based measures of disease prevalence, to a different approach, using a combination of clinical data on diagnosed disease and survey estimates (online supplemental Appendix 8). We find that for diabetes and hypertension, these give very different results, showing the opposite geographical pattern, while for depression, the pattern is more similar between the two methods.

## Discussion

Our analysis indicates relatively high levels of underdiagnosis of diabetes, hypertension and depression in England. For diabetes and depression, there is evidence that diagnosis rates have improved over time. However, this is not the case for hypertension. For hypertension and to a lesser extent diabetes, survey responses indicate people with these conditions in more deprived areas are more likely to be diagnosed than those in less deprived areas. The opposite was true for people experiencing depression. Age and gender appear to be important determinants of whether people with these diseases receive a diagnosis. In particular, younger men with clinical signs of hypertension or diabetes were less likely to report that they were diagnosed. Both younger and older age groups with signs of depression were less likely to be diagnosed compared with middle age groups (30–70).

Our approach to estimating levels of underdiagnosis has several strengths and limitations. As outlined above**,** the survey-based approach has the strength that it is based on direct measurements of clinical signs of disease along with self-reported diagnosis that can be updated annually. Rather than comparing survey-based estimates of total prevalence with diagnosed prevalence from clinical records, we directly estimate the probability of diagnosis based on survey responses related to self-reported clinical diagnosis and clinical measurement of signs of disease for the same individuals. There are, however, various biases that arise from using self-reported measures in survey data. Although we use survey weights in our analysis, overall non-response in these surveys, not accounted for in these weights, could influence the generalisability of the findings. Differential response of self-reported diagnosis may be biased, with some groups being more aware of their clinical diagnosis than others, for example. This could lead to bias if patterns of self-reported diagnosis do not reflect patterns of actual diagnoses by clinicians. Bias may also be introduced if there is non-random measurement error or response in the measures of clinical signs used. For example, previous studies have found that some mental health symptom scales lead to greater false positive rates in relation to detection of clinical depression in more affluent groups compared with more disadvantaged groups.^26^ An additional limitation of the self-reported data is that participants in both HSE and UKLHS were asked to report if they had ‘ever’ been told by a doctor or nurse that they had a condition. We therefore do not have the timing of diagnosis, and so the self-report of a condition may not match an individual’s current diagnosis at the time of the survey’s clinical symptom collection. The large differences we find between approaches just using survey data and an approach using a combination of survey data and area-based measures of prevalence from clinical records suggest that there may be systematic biases either in the survey self-reports of diagnosis or the clinical coding of conditions or both. Further research using individual level healthcare record data linked to survey based biometric information as opposed to self-report diagnoses could further enhance understanding of underdiagnosis patterns.

The use of relatively short screening instruments for identifying people with probable depression may also identify relatively large numbers of people with psychological distress who are not experiencing clinical disease, and the extent to which this is the case may vary by population characteristics.^26^ Blood pressure measurements in the HSE are based on three readings taken at 1 min intervals, which likely overestimates levels of hypertension. Measurements at a single time period lead to relatively high levels of false positives compared with longer-term monitoring of hypertension, and this false positive rate may vary by population characteristics.^27 28^ HbA1c was only measured on one occasion, which may not accurately measure presence of diabetes compared with two abnormal tests that would be used for diagnosis in a clinical setting. A further limitation is that sample sizes and the availability of disaggregate small area population estimates mean that it is only possible to directly estimate levels of underdiagnosis across a small number of population segments.

Several studies have estimated the overall prevalence of diabetes, hypertension and depression, including diagnosed and undiagnosed cases,^4 9 29 30^ some of which present overall national levels of the fraction of these diseases that are undiagnosed. Very few studies, however, have investigated factors associated with levels of underdiagnosis. Two reports^3 31^ investigating the risk factors for undiagnosed hypertension, using similar measures and the same data set (HSE), found that people living *outside* London were more likely to be undiagnosed, similar to our findings. Another study that compared small area estimates of total prevalence modelled from the HSE to clinical records of a number of diagnosed cases from the QOF reported higher levels of underdiagnosis *inside* London.^8^ This reflects our finding that the geographical pattern of estimated underdiagnosis changes markedly from using just survey-based estimates compared with estimates that combine measures of total prevalence from HSE and diagnosed numbers from primary care records. As we found, this reverses the relative levels of underdiagnosis in London compared with the rest of the country. It may be that there are systematic biases in the self-reporting of diagnosis for hypertension and diabetes in HSE for some populations—particularly those in London. Alternatively, it could be that there is underreporting of clinical diagnoses in primary care systems in London. Another study using mortality records to model levels of underdiagnosis also found lower levels of underdiagnoses of hypertension in London.^32^ While one report concludes that lower levels of underdiagnosis may reflect better access to services in London,^31^ inconsistency in findings when applying different methods makes it difficult to infer what underlies these patterns. It could, for example, be that in London more people have had a diagnosis from clinicians other than a National Health Service (NHS) GP, either privately or from outside the UK. Our evidence for the geographical pattern of undiagnosed depression is clearer, regardless of the method applied; we find a particular problem with underdiagnosis in London. Previous studies have noted that prescribing rates for antidepressants are much lower in London than in comparable areas and hypothesised that this is due to poorer access to diagnosis and treatment.^33 34^ The evidence presented here suggests this may be the case.

The higher levels of underdiagnosis for hypertension^3 31^ and depression^35^ that we observe for men compared with women align with other research on gender differences in healthcare access. Women have on average higher rates of primary care consultations than men, particularly between ages 21–39 when women are more likely to have contact with the health system for reproductive or child-rearing reasons.^36^ While this gender difference diminishes slightly after taking consultations for reproductive reasons into account, the increased contact with the health system makes further consultation and diagnosis more likely.^36^ While we do not find an overall gender difference in diabetes underdiagnosis, we do find a lower level for 30–49 year old women, and other studies have reported that women with diabetes were less likely to be undiagnosed.^2 37^ Moreover, there is some evidence from a community survey of over 50s that women are more likely than men to visit the GP when experiencing multiple symptoms.^38^ One hypothesis for the gender difference in undiagnosed depression is that women are more likely to self-report mild–moderate symptoms of depression and therefore are more likely to be diagnosed than men.^35^

The only other study, to our knowledge, that has estimated the fraction of people with depression in England that are diagnosed is the Adult Psychiatric Morbidity Survey (APMS).^4^ This reported lower levels of underdiagnosis than reported here (36% in 2014 compared with 66% in our study). This is probably because the SF-12 is identifying potentially high numbers of people with psychological distress but without diagnosable disease. If we take into account the estimated positive predictive value of 48% for the SF-12 MCS threshold we used (online supplemental Appendix 1), our estimates of underdiagnosis would reduce somewhat to 49% being undiagnosed. This also potentially reflects the underreporting of diagnosed depression in UKHLS, with diagnosed prevalence in the survey being somewhat lower than that reported in the APMS (8% in UKHLS compared with 21% in the APMS). Whilst the 2014 APMS study did not directly estimate risk factors for being undiagnosed, it did find a similar U-shaped relationship with age, with younger and older age groups with symptoms based on the Clinical Interview Schedule-Revised being less likely to have received treatment.^4^ This had, however, changed in the recently published 2023/2024 survey, where unmet needs in younger age groups have apparently reduced, while there remained evidence of undertreatment in older age groups.

Analysis of the 2019 Brazilian National Survey similarly found lowest rates of undiagnosed depression in those aged 36–59, with highest rates in 15–20 year old followed by those aged 60+. Hypothesised reasons in the literature for underdiagnosis at older ages include symptom profile, clinician behaviours, health-seeking behaviours, stigma and access to support.^39 40^ UK results from the 2014 European Health Interview Survey which used the Patient Health Questionnaire (PHQ-8) to assess depressive symptoms found that those aged 75+ had the highest rate of mild depressive symptoms (PHQ-8 score of 5–9); this was under the threshold commonly used for clinical depression, while those aged 45–59 had the highest rates of severe depressive symptoms (PHQ-8 score ≥15).^41^ This may suggest that while older adults are more likely to present depressive symptoms, they may be less likely to reach clinical thresholds for diagnosis. We still, however, see a similar relationship with age when using a lower threshold on the MCS to define more severe clinical signs of depression, suggesting severity of symptom profile does not explain this pattern. The 2023–2024 APMS found that older people were both the least likely age group to receive treatment and also the least likely to report they had sought and not received treatment, suggesting the low treatment levels were due to a lack of help-seeking.^42^

The findings that levels of underdiagnosis for hypertension and, to a lesser extent, diabetes are higher in younger and less deprived groups have been highlighted by others^3^ but are not widely recognised. Results from the 2021 HSE also report a higher proportion of hypertension and diabetes being undiagnosed or untreated in less deprived populations, compared with more deprived populations.^43^ A previous study^31^ found that higher socioeconomic status (education and occupational status) was associated with higher levels of underdiagnosis for hypertension, but only for women. One study using the English Longitudinal Study of Ageing found no relationship between the likelihood of diabetics being diagnosed and social class.^37^ A study by the Office for National Statistics^2^ found that diabetes cases were more likely to be undiagnosed if they were in better general health, which may explain our finding of a slight trend towards higher levels of underdiagnosis in less deprived groups. This pattern may reflect biases in reporting of diagnoses in survey data and needs to be assessed using survey data linked to clinical records. It could also reflect that more deprived populations are more likely to have multiple health conditions and therefore more contact with health services leading to greater opportunities for identifying undiagnosed hypertension and diabetes. One study suggested the higher rates of hypertension underdiagnosis in less deprived women may be due to a lower rate of GP consultations among more affluent women, who also have lower levels of reproductive-related healthcare use than more deprived women (largely due to having fewer children).^31^ A US study found contrasting findings—a higher proportion of hypertension undiagnosed in deprived neighbourhoods,^44^ suggesting that the pattern we observe in the UK of a lower proportion of hypertension undiagnosed in deprived neighbourhoods may relate to more equitable access to healthcare in the UK. If this finding holds up to further scrutiny, it presents a challenge to health inequalities policy. Allocating resources to increase the probability of diagnosis in these populations would lead to greater health benefits in populations with pre-existing better health (eg, less deprived populations)—potentially widening health differences.

The level and pattern of underdiagnosis identified in this study have important implications for policy and practice. While progress has been made in improving the diagnosis of diabetes and depression, as reported in other studies,^37^ we find no evidence of an improved probability of diagnosis with hypertension over time. This is surprising given the focus of national health programmes on the identification and treatment of hypertension, for example, through the NHS’s health inequalities strategy and the NHS health check programme.^45^ These health checks start at age 40; the present study may suggest that screening for hypertension should happen at a younger age, especially in individuals with risk factors such as obesity and harmful alcohol consumption. We need a better understanding of the reasons for the lack of progress over the past decade to inform the current strategy. Our findings suggest further population health benefits could result from the identification and management of hypertension.

Strategies to identify and improve diagnosis of previously undiagnosed chronic diseases should target those populations with higher levels of underdiagnosis. Approaches need to be disease-specific as these patterns differ by disease. While further work is required to understand the causes of underdiagnosis, it is likely that strategies will need to address patient and health system factors, increasing awareness of signs and symptoms as well as developing accessible and culturally appropriate methods for effective case finding. To additionally address health inequalities, undiagnosed case finding should focus on those conditions, such as depression, that are more likely to be undiagnosed in more deprived populations.

## Supplementary material

10.1136/bmjph-2024-001919 online supplemental file 1
