# Supplementary material for: Unveiling the hidden burden: estimating the proportion of undiagnosed depression, hypertension and diabetes – a modelling study using survey data from adults in England, 2011–2019
Source: BMJ Public Health. 2025 Oct 30;3(2):e001919. doi: 10.1136/bmjph-2024-001919 (PMC12581041; doi:10.1136/bmjph-2024-001919)
Supplement: online supplemental file 1 [file bmjph-3-2-s001.docx]

**Supplementary Materials. Unveiling the Hidden Burden: Estimating the Proportion of Undiagnosed Depression, Hypertension and Diabetes across England, 2011-2019.**

# **Appendix 1. Estimates from the Adult Psychiatric Morbidity survey 2007 comparing different thresholds of the SF-12 MCS to a diagnosis of depression defined using the CIS-R.**

Whilst the SF-12 MCS has been used to screen populations for mental health conditions, it does not provide a precise diagnostic definition for depression. The threshold used will influence the sensitivity and specificity of this as a measure of clinical diseases. In supplementary analysis, we therefore compare thresholds of the SF-12 MCS to cases of depression defined using the Clinical Interview Schedule-Revised (CIS-R), which does provide structured diagnostic categories of common mental health disorders, including depression based on the Diagnostic Statistical Manual for Mental Disorder definitions. For this purpose, we used the Adult Psychiatric Morbidity Survey (APMS) 2007,^29^ which included both measures and could therefore be used to compare the MCS to a diagnostic interview, although it is not carried out sufficiently frequently or with a big enough sample for directly estimating annual levels of underdiagnosis for small areas. This analysis shows that using a threshold of ≤ 42 in the MCS gave the same overall level of underdiagnosis nationally (% of disease cases undiagnosed) in the APMS when compared to using the CIS-R measure of depression. Based on this APMS analysis, a threshold of ≤ 42 gives a specificity of the MCS of 98%, a sensitivity of 53%, a positive predictive value (PPV) of 48% and a negative predictive value of 94% when compared to the CIS-R (see appendix 1). This suggests the MCS at this threshold is probably a reasonable reflection of the distribution of clinical signs of depressive disorders. However, to test the sensitivity of our analysis to the threshold set – we replicate the results using a more restrictive threshold of ≤ 30, which gives a slightly higher PPV of 55% in our APMS analysis.

*Figure S1. Estimates from the Adult Psychiatric Morbidity survey 2007 comparing different thresholds of the SF-12 MCS to a diagnosis of depression defined using the CIS-R.*

# **Appendix 2. Definition of diagnosed depression in UKHLS**

The questions used in the UKHLS concerning diagnosis varied depending on the wave. In waves 3-9, respondents were asked:

“Has a doctor or other health professional ever told you that you have any of these conditions?”

“Clinical Depression” was one of the 17 conditions listed on the showcard that respondents used to identify diagnosed conditions.

We defined diagnosed depression cases as those who reported being diagnosed with “clinical depression”

From wave 10 (2017) respondents were asked:

“Has a doctor or other health professional ever told you that you have any of these conditions?”

The showcard then included 18 conditions, but “Clinical Depression” was not present, instead this was replaced with “An emotional, nervous or psychiatric problem”. If they indicated they had been diagnosed with an “An emotional, nervous or psychiatric problem”, they were asked an additional question:

What type of emotional, nervous or psychiatric problem was that?

The showcard then included 6 mental health conditions, including “depression” and “bipolar disorder or manic depression”

We defined depression cases as those who reported being diagnosed with “depression” or “bipolar disorder or manic depression” at this second stage.

# **Appendix 3. Summary of data**

**Table S1. Summary characteristics of Health Survey for England 2011-2019**

|  | Overall |
| --- | --- |
| n | 35,669 |
| **Age group (%)** |  |
| 18_29 | 3595 (10.1) |
| 30_49 | 11826 (33.2) |
| 50_59 | 6697 (18.8) |
| 60_69 | 6903 (19.4) |
| 70_79 | 4678 (13.1) |
| 80_99 | 1970 ( 5.5) |
| **Gender = M (%)** | 15970 (44.8) |
| **Deprivation quintile (%)** |  |
| Q1 Least Deprived | 7967 (22.3) |
| Q2 | 7781 (21.8) |
| Q3 | 7460 (20.9) |
| Q4 | 6572 (18.4) |
| Q5 Most Deprived | 5889 (16.5) |
| **Region (%)** |  |
| East Midlands | 3407 ( 9.6) |
| East of England | 4001 (11.2) |
| London | 3675 (10.3) |
| North East | 3101 ( 8.7) |
| North West | 4691 (13.2) |
| South East | 5986 (16.8) |
| South West | 3926 (11.0) |
| West Midlands | 3561 (10.0) |
| Yorkshire And The Humber | 3321 ( 9.3) |
| **Diagnosed diabetes (%)** |  |
| No diagnosis | 33337 (93.5) |
| Diagnosis | 2326 ( 6.5) |
| Missing | 6 ( 0.01) |
| **Diabetes clinical signs (%)** |  |
| HBA1c<48 mmols | 31952 (89.6) |
| HBA1c>=48 mmols | 2180 ( 6.1) |
| Missing | 1537 ( 4.3) |
| **Any diabetes** |  |
| No diabetes | 31135 (87.3) |
| Diabetes diagnosis or HBA1c>=48 mmols | 2983 ( 8.4) |
| Missing | 1551 ( 4.3) |
| **Hypertension diagnosis (%)** |  |
| No diagnosis | 26522 (74.4) |
| Diagnosis | 9126 (25.6) |
| Missing | 21 ( 0.1) |
| **Hypertension – clinical signs (%)** |  |
| SBP<=140mmHg | 24908 (69.8) |
| SBP>140mmHg | 5676 (15.9) |
| Missing | 5085 (14.3) |
| **Any hypertension** |  |
| No Hypertension | 19642 (55.1) |
| Diagnosed hypertension or SBP>140mmHg | 12131 (34.0) |
| Missing | 3896 (10.9) |

**Table S2. Summary characteristics of Understanding Society UK Longitudinal Household Survey 2011-2019**

|  | Overall |
| --- | --- |
| n | 179,210 |
| **Age group (%)** |  |
| 16_29 | 26326 (14.7) |
| 30_49 | 55567 (31.0) |
| 50_59 | 32382 (18.1) |
| 60_69 | 31491 (17.6) |
| 70_79 | 22897 (12.8) |
| 80_99 | 10547 ( 5.9) |
| **Gender = M (%)** | 77090 (43.0) |
| **Deprivation quintile(%)** |  |
| Q1 Least Deprived | 38346 (21.4) |
| Q2 | 38310 (21.4) |
| Q3 | 36469 (20.3) |
| Q4 | 33313 (18.6) |
| Q5 Most Deprived | 32772 (18.3) |
| **Region (%)** |  |
| East Midlands | 18137 (10.1) |
| East of England | 20498 (11.4) |
| London | 22902 (12.8) |
| North East | 8978 ( 5.0) |
| North West | 23670 (13.2) |
| South East | 28233 (15.8) |
| South West | 20127 (11.2) |
| West Midlands | 18357 (10.2) |
| Yorkshire And The Humber | 18308 (10.2) |
| **Diagnosed depression (%)** |  |
| No diagnosis | 162933 (90.9) |
| Diagnosis | 16055 ( 9.0) |
| Missing | 222 ( 0.1) |
| **Depression symptoms (%)** |  |
| SFS12 MCS>42 | 132576 (74.0) |
| SFS12 MCS<=42 | 35860 (20.0) |
| Missing | 10774 ( 6.0) |
| **Any Depression** |  |
| No depression | 125213 (69.9) |
| Diagnosed depression or SFS12 MCS<=42 | 43951 (24.5) |
| Missing | 10046 ( 5.6) |

# **Appendix 4. Regression model coefficients and goodness of fit tests.**

The main regression model for each condition is a logistic regression of the form

Model 1.

$$\ln\left( p/(1-p) \right)=B_{1}X_{1}+B_{2}X_{2}+B_{3}X_{3}+B_{4}X_{4}+B_{5}X_{5}+$$

$$B_{6}{X_{1}X}_{2}+B_{7}{X_{1}X}_{3}+B_{8}X_{1}X_{4}+B_{9}X_{1}X_{5}$$

$${+B}_{10}{X_{2}X}_{1}+B_{11}{X_{2}X}_{3}+B_{12}X_{2}X_{4}+B_{13}X_{2}X_{5}$$

$${+B}_{14}{X_{3}X}_{1}+B_{15}{X_{3}X}_{2}+B_{16}X_{3}X_{4}+B_{17}X_{3}X_{5}$$

$${+B}_{18}{X_{4}X}_{1}+B_{19}{X_{4}X}_{3}+B_{20}X_{4}X_{2}+B_{21}X_{4}X_{5}$$

$${+B}_{22}{X_{5}X}_{1}+B_{23}{X_{5}X}_{2}+B_{24}X_{5}X_{3}+B_{25}X_{5}X_{4}+C$$

Where $p$ is the probability of an individual not having reported that they have ever been diagnosed, with the model estimated on a subset of the data, where people have either reported they have a diagnosis or have clinical signs of the condition.

$X_{1}$ is the age group of the respondent

$X_{2}$ is the sex of the respondent

$X_{3}$ is the deprivation quintile of the respondent

$X_{4}$ is the region of the respondent

$X_{5}$ is a time trend term for year.

The final model for each condition only includes those interactions that improved model fit based in Akaike information criterion . We used stepwise model selection by Akaike information criterion (AIC) to find the combination of interactions that provides the best fitting model for the data. The full regression output is given in the tables below:

**Table S3: Logistic regression model giving the Odds Ratio of being undiagnosed with Diabetes conditional on having diabetes (diagnosed and undiagnosed).**

| **Characteristic** | **OR** | **95% CI** | **p-value** |
| --- | --- | --- | --- |
| Age group |  |  | 0.5 |
| 18_29 | — | — |  |
| 30_49 | 0.54 | 0.24, 1.29 | 0.2 |
| 50_59 | 0.76 | 0.35, 1.77 | 0.5 |
| 60_69 | 0.76 | 0.35, 1.75 | 0.5 |
| 70_79 | 0.64 | 0.29, 1.47 | 0.3 |
| 80_99 | 0.75 | 0.34, 1.74 | 0.5 |
| Sex |  |  | 0.9 |
| F | — | — |  |
| M | 0.94 | 0.35, 2.58 | 0.9 |
| Deprivation quintile |  |  | 0.035 |
| Q1 Least Deprived | — | — |  |
| Q2 | 1.65 | 0.98, 2.78 | 0.061 |
| Q3 | 1.09 | 0.64, 1.86 | 0.7 |
| Q4 | 1.19 | 0.71, 2.02 | 0.5 |
| Q5 Most Deprived | 0.76 | 0.44, 1.32 | 0.3 |
| Year | 0.98 | 0.90, 1.06 | 0.6 |
| Age group * Sex |  |  | 0.002 |
| 30_49 * M | 2.01 | 0.68, 5.83 | 0.2 |
| 50_59 * M | 0.81 | 0.28, 2.27 | 0.7 |
| 60_69 * M | 0.70 | 0.25, 1.94 | 0.5 |
| 70_79 * M | 0.78 | 0.27, 2.19 | 0.6 |
| 80_99 * M | 0.62 | 0.20, 1.82 | 0.4 |
| Sex * Year |  |  | 0.2 |
| M * Year | 1.04 | 0.98, 1.10 | 0.2 |
| Deprivation quintile * Year |  |  | 0.3 |
| Q2 * Year | 0.92 | 0.83, 1.02 | 0.12 |
| Q3 * Year | 1.00 | 0.91, 1.11 | >0.9 |
| Q4 * Year | 0.96 | 0.87, 1.06 | 0.4 |
| Q5 Most Deprived * Year | 0.99 | 0.90, 1.10 | 0.9 |
| Abbreviations: CI = Confidence Interval, OR = Odds Ratio | | | |

**Table S4: Logistic regression model giving the Odds Ratio of being undiagnosed with hypertension conditional on having hypertension (diagnosed and undiagnosed).**

| **Characteristic** | **OR** | **95% CI** | **p-value** |
| --- | --- | --- | --- |
| Age group |  |  | <0.001 |
| 18_29 | — | — |  |
| 30_49 | 2.05 | 1.33, 3.32 | 0.002 |
| 50_59 | 2.89 | 1.88, 4.65 | <0.001 |
| 60_69 | 2.59 | 1.69, 4.15 | <0.001 |
| 70_79 | 2.15 | 1.40, 3.45 | <0.001 |
| 80_99 | 1.81 | 1.16, 2.93 | 0.011 |
| Sex |  |  | <0.001 |
| F | — | — |  |
| M | 12.7 | 8.07, 20.7 | <0.001 |
| Deprivation quintile |  |  | <0.001 |
| Q1 Least Deprived | — | — |  |
| Q2 | 1.05 | 0.94, 1.17 | 0.4 |
| Q3 | 0.97 | 0.86, 1.08 | 0.6 |
| Q4 | 0.79 | 0.70, 0.89 | <0.001 |
| Q5 Most Deprived | 0.68 | 0.60, 0.78 | <0.001 |
| Region |  |  | <0.001 |
| East Midlands | — | — |  |
| East of England | 0.79 | 0.67, 0.94 | 0.006 |
| London | 0.76 | 0.65, 0.90 | 0.001 |
| North East | 1.04 | 0.85, 1.26 | 0.7 |
| North West | 1.01 | 0.86, 1.18 | >0.9 |
| South East | 0.90 | 0.78, 1.05 | 0.2 |
| South West | 1.01 | 0.86, 1.19 | >0.9 |
| West Midlands | 0.88 | 0.75, 1.04 | 0.14 |
| Yorkshire And The Humber | 0.91 | 0.77, 1.08 | 0.3 |
| Age group * Sex |  |  | <0.001 |
| 30_49 * M | 0.17 | 0.10, 0.27 | <0.001 |
| 50_59 * M | 0.08 | 0.05, 0.13 | <0.001 |
| 60_69 * M | 0.08 | 0.05, 0.12 | <0.001 |
| 70_79 * M | 0.06 | 0.04, 0.10 | <0.001 |
| 80_99 * M | 0.08 | 0.05, 0.13 | <0.001 |
| Abbreviations: CI = Confidence Interval, OR = Odds Ratio | | | |

**Table S5: Logistic regression model giving the OR of being undiagnosed with depression conditional on having depression (diagnosed and undiagnosed).**

| **Characteristic** | **OR** | **95% CI** | **p-value** |
| --- | --- | --- | --- |
| Age group |  |  |  |
| 16_29 | — | — |  |
| 30_49 | 0.41 | 0.32, 0.52 | <0.001 |
| 50_59 | 0.29 | 0.22, 0.38 | <0.001 |
| 60_69 | 0.17 | 0.12, 0.23 | <0.001 |
| 70_79 | 0.45 | 0.31, 0.67 | <0.001 |
| 80_99 | 0.56 | 0.33, 0.95 | 0.028 |
| Sex |  |  |  |
| M | — | — |  |
| F | 0.43 | 0.36, 0.53 | <0.001 |
| Deprivation quintile |  |  |  |
| Q1 Least Deprived | — | — |  |
| Q2 | 1.10 | 0.84, 1.45 | 0.5 |
| Q3 | 1.08 | 0.82, 1.44 | 0.6 |
| Q4 | 1.25 | 0.94, 1.66 | 0.12 |
| Q5 Most Deprived | 1.38 | 1.04, 1.82 | 0.026 |
| Region |  |  |  |
| East Midlands | — | — |  |
| East of England | 0.92 | 0.66, 1.28 | 0.6 |
| London | 1.09 | 0.77, 1.55 | 0.6 |
| North East | 0.61 | 0.41, 0.93 | 0.020 |
| North West | 1.07 | 0.76, 1.49 | 0.7 |
| South East | 0.59 | 0.44, 0.79 | <0.001 |
| South West | 0.69 | 0.49, 0.96 | 0.029 |
| West Midlands | 0.61 | 0.44, 0.85 | 0.004 |
| Yorkshire And The Humber | 0.81 | 0.58, 1.14 | 0.2 |
| Year | 1.00 | 0.97, 1.04 | 0.8 |
| Age group * Sex |  |  |  |
| 30_49 * F | 1.12 | 1.00, 1.25 | 0.049 |
| 50_59 * F | 1.26 | 1.11, 1.42 | <0.001 |
| 60_69 * F | 1.56 | 1.36, 1.79 | <0.001 |
| 70_79 * F | 1.68 | 1.41, 2.00 | <0.001 |
| 80_99 * F | 1.67 | 1.31, 2.14 | <0.001 |
| Age group * Deprivation quintile |  |  |  |
| 30_49 * Q2 | 0.85 | 0.71, 1.02 | 0.073 |
| 50_59 * Q2 | 0.93 | 0.76, 1.14 | 0.5 |
| 60_69 * Q2 | 0.94 | 0.75, 1.16 | 0.6 |
| 70_79 * Q2 | 0.93 | 0.71, 1.21 | 0.6 |
| 80_99 * Q2 | 1.25 | 0.86, 1.81 | 0.2 |
| 30_49 * Q3 | 0.92 | 0.77, 1.10 | 0.4 |
| 50_59 * Q3 | 0.88 | 0.72, 1.08 | 0.2 |
| 60_69 * Q3 | 0.94 | 0.75, 1.17 | 0.6 |
| 70_79 * Q3 | 0.94 | 0.72, 1.23 | 0.7 |
| 80_99 * Q3 | 1.48 | 1.02, 2.13 | 0.038 |
| 30_49 * Q4 | 0.73 | 0.62, 0.88 | <0.001 |
| 50_59 * Q4 | 0.92 | 0.75, 1.12 | 0.4 |
| 60_69 * Q4 | 1.52 | 1.22, 1.90 | <0.001 |
| 70_79 * Q4 | 0.86 | 0.66, 1.12 | 0.3 |
| 80_99 * Q4 | 1.05 | 0.72, 1.53 | 0.8 |
| 30_49 * Q5 Most Deprived | 0.74 | 0.62, 0.88 | <0.001 |
| 50_59 * Q5 Most Deprived | 0.81 | 0.66, 0.98 | 0.030 |
| 60_69 * Q5 Most Deprived | 1.19 | 0.96, 1.48 | 0.11 |
| 70_79 * Q5 Most Deprived | 1.25 | 0.94, 1.67 | 0.12 |
| 80_99 * Q5 Most Deprived | 1.27 | 0.86, 1.89 | 0.2 |
| Age group * Region |  |  |  |
| 30_49 * East of England | 1.02 | 0.79, 1.31 | >0.9 |
| 50_59 * East of England | 1.13 | 0.85, 1.50 | 0.4 |
| 60_69 * East of England | 1.22 | 0.89, 1.67 | 0.2 |
| 70_79 * East of England | 1.04 | 0.70, 1.54 | 0.8 |
| 80_99 * East of England | 0.87 | 0.52, 1.46 | 0.6 |
| 30_49 * London | 1.37 | 1.07, 1.75 | 0.013 |
| 50_59 * London | 1.11 | 0.84, 1.47 | 0.4 |
| 60_69 * London | 1.53 | 1.11, 2.10 | 0.009 |
| 70_79 * London | 1.65 | 1.09, 2.52 | 0.019 |
| 80_99 * London | 5.27 | 2.54, 11.8 | <0.001 |
| 30_49 * North East | 1.63 | 1.21, 2.19 | 0.001 |
| 50_59 * North East | 1.60 | 1.15, 2.22 | 0.005 |
| 60_69 * North East | 1.67 | 1.15, 2.41 | 0.007 |
| 70_79 * North East | 2.16 | 1.37, 3.40 | <0.001 |
| 80_99 * North East | 2.49 | 1.29, 4.89 | 0.007 |
| 30_49 * North West | 1.64 | 1.29, 2.08 | <0.001 |
| 50_59 * North West | 1.41 | 1.08, 1.83 | 0.011 |
| 60_69 * North West | 1.87 | 1.38, 2.52 | <0.001 |
| 70_79 * North West | 1.61 | 1.10, 2.36 | 0.014 |
| 80_99 * North West | 1.90 | 1.13, 3.19 | 0.015 |
| 30_49 * South East | 1.37 | 1.09, 1.72 | 0.007 |
| 50_59 * South East | 1.30 | 1.01, 1.68 | 0.045 |
| 60_69 * South East | 1.53 | 1.15, 2.04 | 0.003 |
| 70_79 * South East | 0.78 | 0.55, 1.12 | 0.2 |
| 80_99 * South East | 1.31 | 0.80, 2.11 | 0.3 |
| 30_49 * South West | 1.51 | 1.18, 1.94 | 0.001 |
| 50_59 * South West | 1.74 | 1.32, 2.30 | <0.001 |
| 60_69 * South West | 1.32 | 0.97, 1.79 | 0.075 |
| 70_79 * South West | 1.06 | 0.72, 1.54 | 0.8 |
| 80_99 * South West | 1.01 | 0.61, 1.66 | >0.9 |
| 30_49 * West Midlands | 2.21 | 1.73, 2.82 | <0.001 |
| 50_59 * West Midlands | 2.17 | 1.65, 2.86 | <0.001 |
| 60_69 * West Midlands | 1.99 | 1.46, 2.71 | <0.001 |
| 70_79 * West Midlands | 2.45 | 1.64, 3.67 | <0.001 |
| 80_99 * West Midlands | 1.24 | 0.75, 2.03 | 0.4 |
| 30_49 * Yorkshire And The Humber | 1.57 | 1.22, 2.01 | <0.001 |
| 50_59 * Yorkshire And The Humber | 1.81 | 1.37, 2.40 | <0.001 |
| 60_69 * Yorkshire And The Humber | 2.16 | 1.59, 2.95 | <0.001 |
| 70_79 * Yorkshire And The Humber | 1.44 | 0.98, 2.14 | 0.066 |
| 80_99 * Yorkshire And The Humber | 4.13 | 2.11, 8.42 | <0.001 |
| Age group * Year |  |  |  |
| 30_49 * Year | 0.97 | 0.96, 0.99 | 0.004 |
| 50_59 * Year | 0.96 | 0.94, 0.98 | <0.001 |
| 60_69 * Year | 0.96 | 0.94, 0.98 | <0.001 |
| 70_79 * Year | 0.91 | 0.88, 0.93 | <0.001 |
| 80_99 * Year | 0.93 | 0.89, 0.97 | <0.001 |
| Sex * Deprivation quintile |  |  |  |
| F * Q2 | 1.23 | 1.08, 1.39 | 0.002 |
| F * Q3 | 1.00 | 0.88, 1.13 | >0.9 |
| F * Q4 | 1.05 | 0.92, 1.19 | 0.5 |
| F * Q5 Most Deprived | 1.24 | 1.09, 1.41 | <0.001 |
| Sex * Region |  |  |  |
| F * East of England | 1.30 | 1.09, 1.55 | 0.004 |
| F * London | 1.04 | 0.87, 1.25 | 0.7 |
| F * North East | 1.07 | 0.87, 1.33 | 0.5 |
| F * North West | 0.95 | 0.80, 1.13 | 0.5 |
| F * South East | 1.27 | 1.08, 1.50 | 0.005 |
| F * South West | 0.94 | 0.79, 1.13 | 0.5 |
| F * West Midlands | 1.23 | 1.02, 1.47 | 0.026 |
| F * Yorkshire And The Humber | 1.06 | 0.88, 1.28 | 0.5 |
| Sex * Year |  |  |  |
| F * Year | 1.02 | 1.00, 1.03 | 0.030 |
| Deprivation quintile * Region |  |  |  |
| Q2 * East of England | 0.61 | 0.46, 0.79 | <0.001 |
| Q3 * East of England | 1.08 | 0.83, 1.41 | 0.6 |
| Q4 * East of England | 0.68 | 0.51, 0.90 | 0.007 |
| Q5 Most Deprived * East of England | 0.54 | 0.41, 0.72 | <0.001 |
| Q2 * London | 0.83 | 0.61, 1.12 | 0.2 |
| Q3 * London | 1.14 | 0.84, 1.55 | 0.4 |
| Q4 * London | 1.04 | 0.77, 1.40 | 0.8 |
| Q5 Most Deprived * London | 0.65 | 0.48, 0.88 | 0.005 |
| Q2 * North East | 0.65 | 0.46, 0.93 | 0.019 |
| Q3 * North East | 1.06 | 0.74, 1.52 | 0.8 |
| Q4 * North East | 0.73 | 0.52, 1.04 | 0.081 |
| Q5 Most Deprived * North East | 0.67 | 0.48, 0.94 | 0.021 |
| Q2 * North West | 0.62 | 0.46, 0.83 | 0.001 |
| Q3 * North West | 0.77 | 0.58, 1.03 | 0.077 |
| Q4 * North West | 0.62 | 0.46, 0.84 | 0.002 |
| Q5 Most Deprived * North West | 0.40 | 0.30, 0.53 | <0.001 |
| Q2 * South East | 0.89 | 0.70, 1.13 | 0.3 |
| Q3 * South East | 0.83 | 0.65, 1.06 | 0.13 |
| Q4 * South East | 0.53 | 0.41, 0.68 | <0.001 |
| Q5 Most Deprived * South East | 0.50 | 0.38, 0.65 | <0.001 |
| Q2 * South West | 0.74 | 0.56, 0.97 | 0.028 |
| Q3 * South West | 1.14 | 0.87, 1.50 | 0.3 |
| Q4 * South West | 0.83 | 0.62, 1.10 | 0.2 |
| Q5 Most Deprived * South West | 0.49 | 0.37, 0.65 | <0.001 |
| Q2 * West Midlands | 0.98 | 0.74, 1.31 | >0.9 |
| Q3 * West Midlands | 0.65 | 0.49, 0.87 | 0.003 |
| Q4 * West Midlands | 0.48 | 0.36, 0.64 | <0.001 |
| Q5 Most Deprived * West Midlands | 0.59 | 0.44, 0.78 | <0.001 |
| Q2 * Yorkshire And The Humber | 0.71 | 0.53, 0.95 | 0.020 |
| Q3 * Yorkshire And The Humber | 0.72 | 0.53, 0.96 | 0.027 |
| Q4 * Yorkshire And The Humber | 0.84 | 0.62, 1.14 | 0.3 |
| Q5 Most Deprived * Yorkshire And The Humber | 0.55 | 0.41, 0.73 | <0.001 |
| Deprivation quintile * Year |  |  |  |
| Q2 * Year | 1.00 | 0.98, 1.02 | 0.8 |
| Q3 * Year | 1.00 | 0.98, 1.03 | 0.7 |
| Q4 * Year | 1.03 | 1.00, 1.05 | 0.024 |
| Q5 Most Deprived * Year | 1.02 | 1.00, 1.04 | 0.058 |
| Region * Year |  |  |  |
| East of England * Year | 0.98 | 0.95, 1.01 | 0.11 |
| London * Year | 0.95 | 0.93, 0.98 | 0.002 |
| North East * Year | 1.00 | 0.97, 1.04 | 0.9 |
| North West * Year | 0.99 | 0.96, 1.01 | 0.3 |
| South East * Year | 1.01 | 0.98, 1.03 | 0.7 |
| South West * Year | 0.97 | 0.94, 1.00 | 0.032 |
| West Midlands * Year | 1.01 | 0.98, 1.04 | 0.7 |
| Yorkshire And The Humber * Year | 0.99 | 0.96, 1.02 | 0.4 |
| Abbreviations: CI = Confidence Interval, OR = Odds Ratio | | | |

Figure S2 show the goodness of fit tests for each model and calibration plots. The Hosmer-Lemeshow test evaluates the null hypothesis is that the logistic regression model is a good fit of the data. For each of the models the null hypothesis is not rejected at the 5% level , as p>0.05

**Figure S2. Calibration plots and Hosmer-Lemeshow Goodness-of-Fit Tests for the three logistic regression models.**


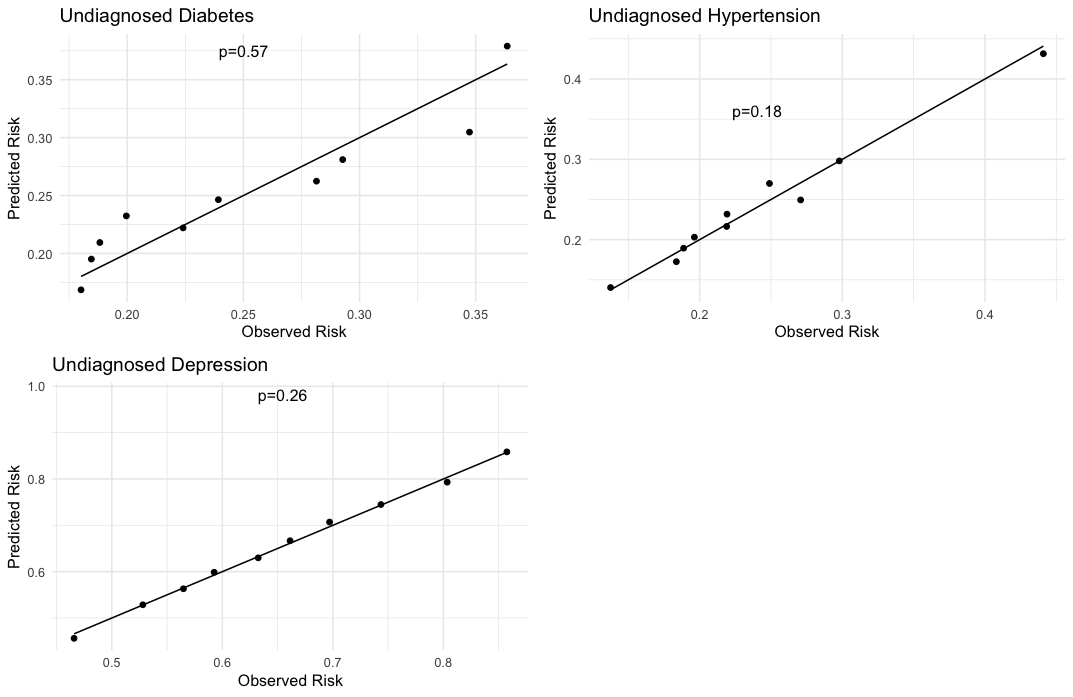


# **Appendix 5. Probability of people with depression being undiagnosed by deprivation and region**

**Figure S3. Mean predictions of the probability of people with depression being undiagnosed by deprivation and region 2011 and 2019.**

# ***Appendix 6.* Using a MCS threshold of <=30 to define probably cases of disease.**

**Figure S4. Mean predictions of the probability of people with depression being undiagnosed by deprivation age and sex 2011 and 2019 – using a MCS threshold of <=30 to define probably cases of disease.**

# **Appendix 7. Microsimulation process.**

**Step1.**

We used the Office for National Statistics mid-year population statistics^24^ to construct a population microdataset for all ~38 million people in England in 2011 and 2019, for each age group (18-29, 30-49, 50-59, 60-69, 70-79, 80+), and sex (male / female), within small neighbourhoods (Lower Super Output Areas), with their associated level of deprivation (IMD quintile), nested within CCGs, that lie within 9 English Regions. This provides a table with ~ 76 (38*2) million records giving the population distribution across age, gender, deprivation and small geographical areas.

**Step 2.**

Initially we need the distribution of disease prevalence (diagnosed and undiagnosed) across this population estimated from our survey data. To do this we estimate an additional logistic regression model using the same procedure as our main model (model 1), however in this case the main outcome is the probability of having either a diagnosis or clinical signs ($p$ =1) or having no diagnosis or clinical signs ($p$ =0). Based on the predicted probability from this model of condition prevalence for each population segments in our micro dataset in step 1, we create a flag (0/1) for each of the 76 million people in our population microdataset drawn from a binomial distribution indicating whether they have each of the 3 conditions.

**Step 3.**

Based on the predicted probability from our main model indicating the probability of being undiagnosed (conditional on having the condition) we create an additional flag (0/1) drawn from a binomial distribution for each of the individuals in our population microdataset, that are flagged with having the disease, indicating whether they are undiagnosed (1) or diagnosed (0).

**Step 4.**

We aggregate up from this individual level population microdataset, to the appropriate ghelath geography (in this case CCG) summing the number with each condition (diagnosed / undiagnosed) and the number undiagnosed, then calculate the proportion undiagnosed. undiagnosed for each CCG.

# **Appendix 8. Comparing the survey based estimates to approach using a combination of clinical data on diagnosed disease and survey estimates.**

We compared our main estimates that only use survey-based measures of disease prevalence (i.e % undiagnosed = survey-based estimates of undiagnosed/ survey-based estimates of total disease prevalence] ), to a different approach using a combination of clinical data on diagnosed disease and survey estimates (i.e % undiagnosed = 100 – [recorded diagnosed prevalence in primary care records / survey-based estimates of total disease prevalence]. The number of people recorded in primary care data as diagnosed with each disease was sourced from the data reported in the Quality and Outcomes Framework (QOF).^19^

*Figure S5. 2019 estimates of percentage of conditions undiagnosed by CCG (darker colour indicates higher % undiagnosed) comparing survey-based only estimates with survey and QOF-based estimates. Missing values (NAs), are for those CCGs for which data on the % diagnosed was not available through the Quality and Outcomes Framework. The categories (bins) used in the colour scheme are set at quintiles of the distribution for each dataset.*
